# Brain–computer interface treatment for gait rehabilitation in stroke patients

**DOI:** 10.3389/fnins.2023.1256077

**Published:** 2023-10-18

**Authors:** Marc Sebastián-Romagosa, Woosang Cho, Rupert Ortner, Sebastian Sieghartsleitner, Tim J. Von Oertzen, Kyousuke Kamada, Steven Laureys, Brendan Z. Allison, Christoph Guger

**Affiliations:** ^1^g.tec Medical Engineering Spain SL, Barcelona, Catalonia, Spain; ^2^g.tec Medical Engineering GmbH, Schiedlberg, Austria; ^3^Department of Neurology 1, Kepler Universitätsklinik, Linz, Austria; ^4^Department for Neurosurgery, Asahikawa Medical University, Asahikawa, Japan; ^5^Hokashin Group Megumino Hospital, Sapporo, Japan; ^6^Coma Science Group, GIGA Consciousness Research Unit, University and University Hospital of Liège, Liège, Belgium; ^7^CERVO Brain Research Center, Laval University, Québec, QC, Canada; ^8^Consciousness Science Institute, Hangzhou Normal University, Hangzhou, Zhejiang, China; ^9^Department of Cognitive Science, University of California, San Diego, La Jolla, CA, United States

**Keywords:** brain–computer interfaces, BCI, stroke, neurorehabilitation, functional electrical stimulation, lower limb, 10 Meter Walking Test, gait

## Abstract

The use of Brain–Computer Interfaces (BCI) as rehabilitation tools for chronically ill neurological patients has become more widespread. BCIs combined with other techniques allow the user to restore neurological function by inducing neuroplasticity through real-time detection of motor-imagery (MI) as patients perform therapy tasks. Twenty-five stroke patients with gait disability were recruited for this study. Participants performed 25 sessions with the MI-BCI and assessment visits to track functional changes during the therapy. The results of this study demonstrated a clinically significant increase in walking speed of 0.19 m/s, 95%CI [0.13–0.25], *p* < 0.001. Patients also reduced spasticity and improved their range of motion and muscle contraction. The BCI treatment was effective in promoting long-lasting functional improvements in the gait speed of chronic stroke survivors. Patients have more movements in the lower limb; therefore, they can walk better and safer. This functional improvement can be explained by improved neuroplasticity in the central nervous system.

## 1. Introduction

Stroke is one of the main causes of mortality and long-term disability worldwide. Functional deficit of the lower limb is the most common paresis after a stroke. Stroke patients rarely fully recover after months or even years of therapy and other treatment, leaving them with permanent impairment. Many of these patients never regain the ability to walk well enough to perform all their daily activities (Hesse et al., 2008; Mehrholz et al., 2017). Gait recovery is one of the major therapy goals in rehabilitation programs for stroke patients and many methods for gait analysis and rehabilitation have been developed (Mehrholz et al., 2017). Weakened muscle tone is another common challenge in motor rehabilitation. Therapies such as active foot drop exercises, electromechanically assisted therapy and treadmill therapy are usually limited to patients with mild or moderate impairment (Mills et al., 2011; Mehrholz et al., 2017).

A 2018 study (Mehrholz et al., 2018) conducted a network meta-analysis based on 95 publications out of 44.567 that were considered. In this study, 4.458 patients were included, and the effectiveness of the most common interventions for gait rehabilitation after stroke was analyzed. The interventions where classified in five groups: (1) No walking training, (2) Conventional walking training (walking on the floor, preparatory exercises in a sitting position, balance training etc. without technical aids and without treadmill training or electromechanical-assisted training), (3) Treadmill training without or with body-weight support, (4) Treadmill training with or without a walking speed paradigm, (5) Electromechanical-assisted training with end-effector devices or exoskeletons. For the primary endpoint of walking speed, end-effector-assisted training (EGAIT_EE) achieved significantly greater improvements than conventional walking rehabilitation (mean difference [MD] = 0.16 m/s, 95% CI = [0.04, 0.28]). None of the other interventions improved walking speed significantly.

Functional electrical stimulation (FES) has also been used in motor rehabilitation therapy over the last few decades. Passive FES therapy can reduce muscle spasms and shorten the term of motor recovery (Hong et al., 2018). Passive therapies such as continuous passive motion or cycling therapy have been employed for patients and showed functional improvements in previous studies (Janssen et al., 2008; Yeh et al., 2010; Ambrosini et al., 2011). However, they do not include devices or systems to monitor the patient’s active engagement in the therapy.

Today, Brain-Computer Interfaces (BCIs) can provide an objective tool for measuring Motor Imagery (MI), creating new possibilities for “closed-loop” feedback (Wolpaw and Wolpaw, 2012). Closed-loop feedback depends on sensing the desired mental activity and is possible with MI-based BCIs, which could significantly improve rehabilitation therapy outcomes (Ortner et al., 2012; Cho et al., 2016; Cantillo-Negrete et al., 2018; Irimia et al., 2018).

MI-based BCIs have been employed in rehabilitation training for stroke patients to fill the gap between patients’ expectations and therapy outcomes. In conventional rehabilitation therapies, patients are often asked to try to move the paretic limb, or to imagine moving it, while a FES, physiotherapist and/or robotic device helps them to perform the desired movement. Their feedback is often provided when the users are not performing the required mental activity. There is no objective way to determine whether patients who cannot move are actively performing the desired motor imagery (MI) task and thus producing concordant neural activation. Its efficacy has been shown in multiple studies implementing exoskeleton, orthosis or robots which induce passive movement of their affected limbs (Ramos-Murguialday et al., 2013; Ono et al., 2014; Ang et al., 2015). During repetitive neurofeedback training sessions, even patients with severe impairment could complete the sensorimotor loop in their brains linking coherent sensory feedback with motor intention (Cho et al., 2016; Pichiorri et al., 2017; Irimia et al., 2018).

This concurrent sensory feedback with motor intention is an important factor for motor recovery (Ortner et al., 2012; Bolognini et al., 2016; Pichiorri et al., 2017; Cantillo-Negrete et al., 2018; Irimia et al., 2018). Concurrent feedback based on users’ intention may help them learn mental strategies associated with movement and BCI use, which can affect results (Neuper et al., 2005; Neuper and Allison, 2014). Neural networks are strengthened when the presynaptic and postsynaptic neurons are both active. In conventional therapies, when patients receive feedback while they are not performing MI, these two neuronal networks are not simultaneously firing. This dissociation between motor commands and sensory feedback may explain why the therapy does not significantly induce the reorganization of the patients’ brains around their lesioned area. Non-simultaneous, dissociated feedback cannot underlie the Hebbian learning between two neuronal populations that underlies the desired improvements from rehabilitation (Mayford et al., 2012; Wolpaw and Wolpaw, 2012). Thus, conventional therapies may sometimes fail because they rely on open-loop feedback.

This clinical trial investigated the impact of combining BCI technology with MI and FES feedback for motor recovery of the lower limbs. The patients’ real-time sensory feedback depended on their movement intention. We explore the relationship between the proposed rehabilitation method and rehabilitation results, including changes in walking speed. Patients who use the training mode may have better motor outcomes, and these outcomes will be compared with those from patients who had EGAIT_EE therapy.

## 2. Methods

### 2.1. Participants and study design

The study was approved by the Ethikkommission des Landes Oberösterreich (Nr. 1,126/2020) and the Bundesamt für Sicherheit im Gesundheitswesen (clinical trial number 101210314) in Austria. Each participant provided written informed consent before the pre-assessment. No adverse events were reported during the entire study period.

Each participant received 3 months of BCI-supported MI training with 3 weekly sessions, 25 sessions in total. Two pre-assessments (Pre1 and Pre2) and three post-assessments (Post1, Post2, and Post3) were performed by two certified physiotherapists and were evaluated by the research team. Pre1 and Pre2 were scheduled 1 month and a few days before the intervention (respectively), while Post1, Post2, and Post3 were carried out a few days, 1 month, and 6 months after the intervention (respectively).

### 2.2. Inclusion and exclusion criteria

The following inclusion criteria were used when recruiting patients: understand written and spoken instructions; at least 10 days after stroke onset; a restriction of the lower extremities that prevents activities of daily life; stable neurological status; willing to participate in the study and to understand and sign the informed consent; available to attend meetings; be able to provide their diagnosis in detail including brain images; can perform 10 Meter Walking Test.

The patients’ recruitment used the following exclusion criteria: ossification; contraction or stiffness of the wrist or ankle joint; metal (e.g., jewelry, piercings, buckles, surgical surface staples) in the target stimulation area; strong visual or auditory deficits that could prevent them from following the tasks; had a brain stem stroke; unable to tolerate cutaneous electrical stimulation; implanted medical devices such as pacemakers; implanted metallic fragments in the extremities which can limit the use of FES; cerebellar lesions; multiple stroke history; elevated intracranial pressure; pronounced hemi-neglect; uncontrolled epilepsy or seizures; under the influence of anesthesia or similar medication; fractures or lesions in the stimulated extremities; severe lung diseases, infections, renal insufficiency, liver damage, heart diseases: severe pusher syndrome; significant circulatory disturbances of the stimulated extremities; inability to independently maintain a seated position (without assistance) for about 60 min; sensory disorders which can significantly affect the patient’s ability to feel pain and react to unsuitable proprioceptive stimuli; diseases of the peripheral nervous system affecting the upper or lower limbs; botulinum-toxin treatment of the paretic lower limb during this study; cognitive impairments that could limit understanding of task instructions (MOCA ≤22).

### 2.3. Subjects

Twenty-five stroke patients were enrolled in this study. Three of them withdrew from the study after the Pre2 assessment. This analysis is based on the 22 remaining patients. Thirteen were males and nine females. The mean age was 53.79 years (SD = 17.22). The median time since the stroke was 47.63 months, IQR = [26.7–99.66]. The time since the stroke ranged between 7 months and 397 months. Twenty-one patients were in the chronic phase, and only 1 was in the subacute phase.

The functional measures recorded in Pre1 and Pre2 assessment did not show any significant differences, indicating that the patients’ functional baseline was stable before the BCI treatment.

### 2.4. Functional and behavioral assessment

The following personal data were recorded for each participant: date of birth; sex; contact information; profession; hobbies, medical history and diagnosis; and previous and current rehabilitation if available.

A series of functional and behavioral scales were administered in pre- and post-assessments. These scales were used to evaluate each patient’s performance in the following five spheres: (1) Gait speed, (2) Balance and gait quality, (3) Range of motion and muscular balance, (4) Motor function, and (5) Cognition and daily living activities. The assessments were conducted by a qualified healthcare professional with expertise in stroke rehabilitation.

#### 2.4.1. Gait speed

The primary measure of this study is the gait speed assessed by the 10 Meter Walking Test (10MWT). The 10MWT assesses walking speed in meters per second over a short duration and is one of the most common ways to evaluate the functional mobility, gait, and balance for lower limb therapy (Wade, 1992). In this test, the individual walks 10 meters without physical assistance. The first and last two meters are not considered to provide time for acceleration and deceleration, and the time to walk the intermediate six meters is measured. Assistive devices can be used but should be kept consistent and documented from test to test. This test is repeated 3 times for more robust measurements.

#### 2.4.2. Balance and gait quality

Timed Up and Go (TUG) assesses the time that patients need to get out of a chair, walk 3 meters, turn around, walk back 3 meters, and sit down again. This measure is related with the coordination and balance (Bohannon, 2006).

The Berg Balance Scale (BBS) was designed to assess static balance and risk to fall. Scores on the BBS range from 0 to 56. 0 and 56 indicate low and high level of function and balance, respectively.

The Functional Ambulation Classification (FAC) categorizes patients according to basic motor skills necessary for functional ambulation. Patients are classified into six different classes: (1) Non-functional, (2) Dependent Level III, (3) Dependent Level II, (4) Dependent with supervision, (5) Independent, level surfaces only, and (6) Independent.

#### 2.4.3. Range of motion and muscular balance

We used the Modified Ashworth Scale (MAS) to assess spasticity, in which low punctuations reflect less spasticity (Meseguer-Henarejos et al., 2018). The MASAnkle was used to test ankle spasticity and the MASKnee scale tested knee spasticity.

The passive and active range of motion (ROMp and ROMa respectively) of the ankle and the knee movements were analyzed using a digital goniometer. The same starting position was used through all measurements in order to keep consistent results and detect changes in mobility.

Muscle strength of the ankle and the knee was assessed by the Manual Muscle Test (MMT), where high values are related to high muscle strength and low values to muscle weakness.

#### 2.4.4. Motor function

The Fugl Meyer Assessment for the Upper Extremity and Lower Extremity (FMA-UE and FMA-LE) was used to evaluate the motor impairment. FMA-UE ranges from 0 to 66 points, and FMA-LE from 0 to 34. The score reflects impairment in limb functions, with lower scores corresponding to greater impairment, and is often used to assess the damage resulting from stroke and progress during therapy (Woytowicz et al., 2017).

#### 2.4.5. Cognition and daily living activities

The Barthel Index (BI) is a questionnaire designed to test the patient’s ability to perform daily living activities (Quinn et al., 2011).

We used the Stroop Color-Word Test (SCWT) and the Montreal Cognitive Assessment (MOCA) for cognitive assessment (under academic license), both tests were in paper version. The SCWT entails three different cards, each with a 10 × 10 matrix of words of color names, and the patient is asked to read as many words as possible in 45 s (Stroop, 1935). The first card is printed in black, the second card contains words printed in the same color (such as the word “BLUE” printed in blue), and the third card has words printed in a different color (such as the word “BLUE” printed in red). People with some types of cognitive dysfunctions will be able to read fewer words than healthy persons. The MOCA scale is widely used to assess the cognitive state of neurologic patients (Koski, 2013). This scale has 8 parts, and the total score ranges from 0 points to 30 points. Higher scores indicate better cognitive function and a MOCA score above 25 points is considered normal.

### 2.5. BCI system description

**Figure 1:**
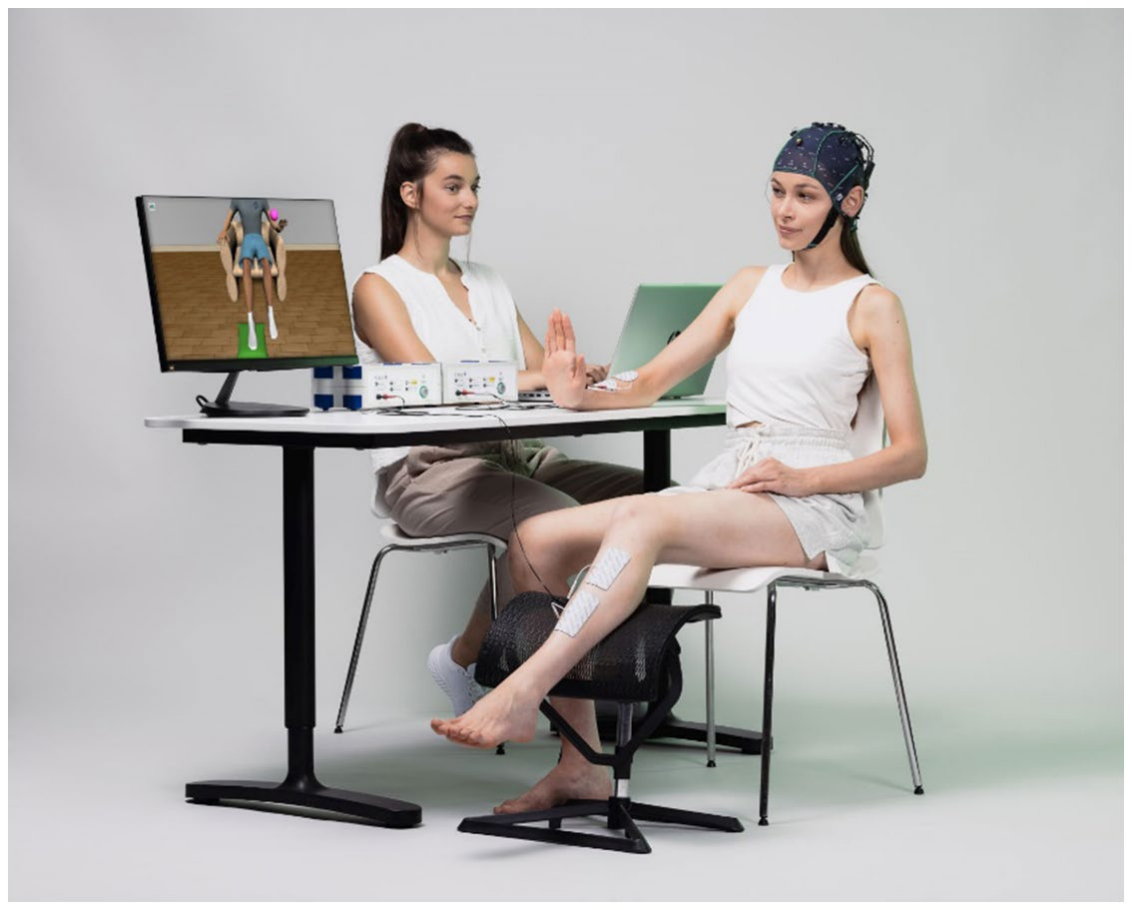
This photograph shows components of the BCI system used in this study, including a monitor with an avatar to instruct the patient and provide visual feedback. The EEG system measures the brain activity that the BCI analyzes in real-time. As soon as the BCI system detects foot or hand movement imagination, the avatar moves its foot or hand while the FES activates to produce the movement.

Patients were seated in a comfortable chair in front of an LCD screen with the arm resting on a desk and the leg on a support chair. Patients wore EEG caps with 16 active electrodes (g.Nautilus PRO, g.tec medical engineering GmbH, Austria). EEG electrode positions were FC3, FCz, FC4, C5, C3, C1, Cz, C2, C4, C6, CP3, CP1, CPz, CP2, CP4 and Pz according to the international 10/20 system. A reference electrode was placed on the right earlobe and a ground electrode at AFz (see Figure 1).

One pair of FES pads were placed on the skin over both the ankle and wrist extensors. The two FES devices (g.Estim FES, g.tec medical engineering GmbH, Austria) were set to a frequency of 50 Hz and a rectangular pulse width of 300 μs for the wrist and 400 μs for the ankle. The stimulation amplitude (in mA) was adjusted to find the optimal movement produced by electrical stimulation in both the healthy and affected limbs without inducing pain or spasms.

All participants were instructed to imagine dorsiflexion of the paretic ankle vs. opposite wrist based on the system indications. This is a type of MI task. Each recording session was divided in 3 runs with 40 trials for each limb type (wrist or ankle). Hence, each session had 240 MI trials. Each session lasted about 1 h, including time for preparation and cleaning.

**Figure 2:**
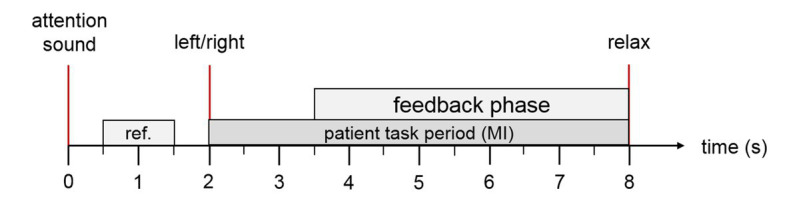
Trial description. The patient hears an attention sound at trial onset. At second 2, the system presents an arrow on the computer screen to instruct the patient to imagine ankle or hand movement (through the left/right cue) and a corresponding verbal instruction in the patient’s native language. During the feedback period, the FES and the virtual avatar are activated if the MI was classified correctly. At second 8, the patient hears a relax command.

The MI tasks were presented in a pseudo-random order with inter-trial intervals of 1 s. Figure 2 depicts the timing of each trial. Patients were first cued to the start of a trial with an attention beep. Two seconds later, an animated arrow in the avatar window pointed either left or right to instruct patients to imagine moving the ankle or hand. At the same time, an auditory instruction cued the patient to imagine either ankle or hand movement during that trial. During the feedback phase, the FES and avatar were triggered when the system detected MI of the correct limb. If no MI was detected, feedback was deactivated. Feedback was updated five times per second.

### 2.6. Signal processing

EEG signals were sent from a biosignal amplifier and were bandpass filtered (4th order Butterworth filter) between 8 and 30 Hz. Then, common spatial patterns (CSP) were applied to transform the data to a new matrix with minimal variance of one class and maximal variance of the other class (Blankertz et al., 2008). Each class reflects the MI of the cued limb versus the MI of the other limb. The CSP method calculated a 16 × 16 projection matrix from 16 EEG channels for each left and right trial *X*. This matrix is a set of spatial patterns that may reflect regional cortical activation during hand MI. The decomposition of a trial is written as *Z = WX*. This transformation projects the variance of *X* onto the rows of *Z* and results in 16 new time series. The columns of A = *W^−1^* are a set of CSPs and are time-invariant EEG distributions. The variance for left trials is largest in the first row of *Z* and decreases with the subsequent rows. The opposite occurs in a trial with right trials. The variances were extracted as reliable features of the newly calculated 16 time series for the binary classification (left vs. right).

According to Mueller-Gerking’s work, the optimal number of CSPs should be four (to reduce the dimensionality of EEG) (Muller-Gerking et al., 1999). Using an artifact corrected training set, *XT*, only the first and last two rows (*p = 1, 2, 15, and 16*) of *W* were used to process new input *X*. Then, the variance (*VARp*) of the time series was calculated for a time window *T*. After normalizing and log-transforming, four feature vectors were obtained via equation 1.


(1) $$f_{p}=\log\left(\frac{VAR_{p}}{\sum_{p=1}^{4}VAR_{p}}\right)$$

A linear discriminant analysis (LDA) classified each trial as either left or right MI. When the input signals were correctly classified according to the assigned task, the feedback devices were triggered. This online classification and control of the FES and avatar were updated every 20 ms.

We estimated offline classification accuracy via a 10-fold cross validation. This refers to partitioning a sample of movements into 10 complementary subsets and validating the analysis on one subset (called the validation set or testing pool) and training the CSPs and classifier on the other subsets (called the training pool).

The accuracy was calculated (in steps of half a second) for all trials in the testing pool within a 4.5 s time window beginning 1.5 s after the attention beep and ending with the end of the trial. For each step and each single trial, the classification result is either 100% or 0%. The accuracy of all trials of the test pool is then averaged for each single step, resulting in accuracy levels ranging between 0 and 100%. After averaging all 10 repetitions of the cross validation, the maximum value during the feedback phase was noted as the session accuracy.

### 2.7. Statistical analysis methods

The software used for the statistical analysis was MATLAB R2020a and RStudio (R version 4.0.3 and RStudio version 2022.02.4). We designated the mean of Pre1 and Pre2 as the baseline value for each outcome measure [Baseline = (Pre1 + Pre2)/2]. Post-assessment was the outcome measure after completion of the 25 training sessions. The primary and secondary outcomes were statistically analyzed after a normal distribution was determined with the Shapiro–Wilk test. The significance threshold was set to α = 0.05. The statistical test was chosen according to the normality of the sample, the homogeneity of variance (Levene’s or Brown-Forsythe test of equal variance) and sample size. Descriptive statistics will be shown as mean and the standard deviation (SD), or the median with the inter-quartile rate (IQR) of 0.25 and 0.75.

A two-tailed paired sample *t*-test or a Wilcoxon signed rank test was used to investigate the outcome of changes between two different assessments in the same group of patients.

For multiple comparisons, *p*-values were corrected using the False Discovery Rate (FDR) described by Benjamini and Hochberg (1995), which explains that adjusted *p*-values can be greater than 1. All *p*-values greater than 1 were converted to 1. The *p*-values below 0.05 that are shown in the results tables are marked in red color.

First, we analyzed the functional improvement after the BCI therapy using paired comparison (*t*-test or Wilcoxon signed rank test) between PRE values and Post1 values. The second step was to analyze the middle-term effects 1 month after therapy by comparing Post1 vs. Post2, and long-term effects 6 months after the therapy by comparing Post1 vs. Post3.

### 2.8. Ethics for re-use

Written informed consent was obtained from the individual (s) for the publication of any potentially identifiable images or data included in this article.

## 3. Results

Twenty-seven patients were assessed for eligibility. Two of these patients were immediately excluded because their stroke location did not meet the inclusion criteria. The other twenty-five patients were assigned to the intervention group (BCI group). Three patients dropped-out from the study. One of those three patients decided to not continue in the study because of a loss of interest. The other two patients could not finish the study because they lacked transportation to attend the sessions. Hence, twenty-two patients finished the BCI sessions, and only results from these patients were analyzed further.

### 3.1. Functional improvement after BCI therapy

The results in this section summarize differences from the Baseline to Post1 assessments across different tests (please see Table 1). We used the Wilcoxon signed rank test or paired t-test for statistical analysis, depending on whether or not the data presented a normal distribution. The improvement of each scale is presented using the median and IQR, and the mean and SD are also provided if differences are significant.

**Table 1:** Summary of the functional improvement after BCI treatment.

Scale	*n*	Baseline Median [IQR]	Post1 Median [IQR]	Δ Median [IQR]	Δ Mean (SD)	*p*
BI	22	90 [80 to 90]	90 [81.25 to 95]	1.25 [0 to 5]	2.73 (SD = 3.26)	0.021
FMA-UE m	22	22.75 [16.25 to 32.75]	24 [18 to 37]	0.75 [−0.38 to 5.62]	2.43 (SD = 4.5)	0.103
FMA-LE m	21	24 [20.5 to 25.5]	25 [22 to 27]	1 [0 to 2.5]	1.1 (SD = 2.68)	0.166
MOCA	20	26 [22.88 to 28.25]	27 [24 to 30]	0.5 [−0.5 to 1.62]	0.82 (SD = 1.58)	0.126
SCWT Word	21	73 [46 to 96]	80 [47 to 98]	3 [−1 to 6]	4.67 (SD = 7.64)	0.044
SCWT Color	21	68 [48 to 93]	65 [50 to 92]	1 [−2 to 6]	1.74 (SD = 6.8)	0.383
SCWT ColorWord	21	24 [15.5 to 30]	26 [18 to 34]	1.5 [0 to 6]	2.9 (SD = 4.06)	0.024
MAS knee	22	0 [0 to 1]	0 [0 to 1]	0 [0 to 0]	−0.2 (SD = 0.47)	0.195
MAS ankle	22	3 [1 to 3]	2.5 [1 to 3]	0 [−1 to 0]	−0.42 (SD = 0.55)	0.038
ROM passive Ankle DF	22	24.75 [19.35 to 31.26]	30.05 [21.33 to 37.25]	4.32 [1.16 to 10.09]	5.67 (SD = 7.89)	0.023
ROM passive Ankle_FL	22	15.05 [8.59 to 21.1]	19.15 [12.92 to 22.73]	2.25 [−0.94 to 5.88]	2.01 (SD = 6.53)	0.281
ROM passive Knee FL	21	128.9 [124.1 to 133.95]	135.3 [126.6 to 139.7]	2.95 [−1 to 9.25]	7.35 (SD = 15.2)	0.043
ROM active Ankle DF	22	7.7 [1.63 to 18.41]	17.7 [2.08 to 30.6]	5.95 [0.45 to 8.64]	7.02 (SD = 7.27)	0.008
ROM active Ankle FL	22	5.55 [3 to 9.18]	10 [3.7 to 15.6]	2.13 [0 to 5.82]	3.7 (SD = 5.84)	0.031
ROM active Knee FL	21	114.6 [109.2 to 118.1]	117.1 [112 to 124.6]	2.5 [−1.1 to 9.5]	6.21 (SD = 12.96)	0.126
MMT Ankle DF	22	6 [4 to 7]	7 [4.5 to 9]	0 [0 to 1]	0.73 (SD = 1.03)	0.031
MMT Ankle FL	22	6 [4 to 7.75]	7.5 [4.25 to 9]	0.25 [0 to 1]	0.84 (SD = 1.18)	0.024
MMT Knee EX	22	8.25 [6.25 to 9]	9 [8 to 10]	0 [0 to 1]	0.68 (SD = 0.91)	0.031
MMT Knee FL	22	9 [8 to 10]	10 [9 to 10]	0 [0 to 1]	0.34 (SD = 0.56)	0.068
TUG	21	14.09 [13.5 to 28.97]	11.69 [11 to 27]	−2.59 [−3.62 to −1.97]	−5.73 (SD = 8)	< 0.001
10MWT SS	22	8.5 [6.8 to 28.91]	6.81 [6.03 to 18.54]	−1.58 [−2.23 to −0.49]	−3.89 (SD = 6.45)	< 0.001
10MWT FV	22	5.78 [5.15 to 26.08]	5.14 [4.56 to 15.09]	−0.99 [−3.9 to −0.46]	−4.75 (SD = 9.09)	0.010
BBS	22	51.25 [28.25 to 54.75]	52.5 [31.75 to 55]	0 [0 to 1.75]	0.61 (SD = 2.18)	0.294
FAC	22	5 [4.12 to 6]	6 [5 to 6]	0 [0 to 0.88]	0.3 (SD = 0.45)	0.053

Gait function was mainly assessed by 10MWT and the balance and coordination by TUG, all of which show some significant improvement after the therapy.

### 3.2. Gait speed

The primary measure of this study is the 10MWT. This test has two different parameters: Self-Selected Velocity (10MWT-SS) and Fast Velocity (10MWT-FV). The results can be presented based on the time (s) or speed (m/s).

#### 3.2.1. Self-selected velocity (10MWT-SS)

**Figure 3:**
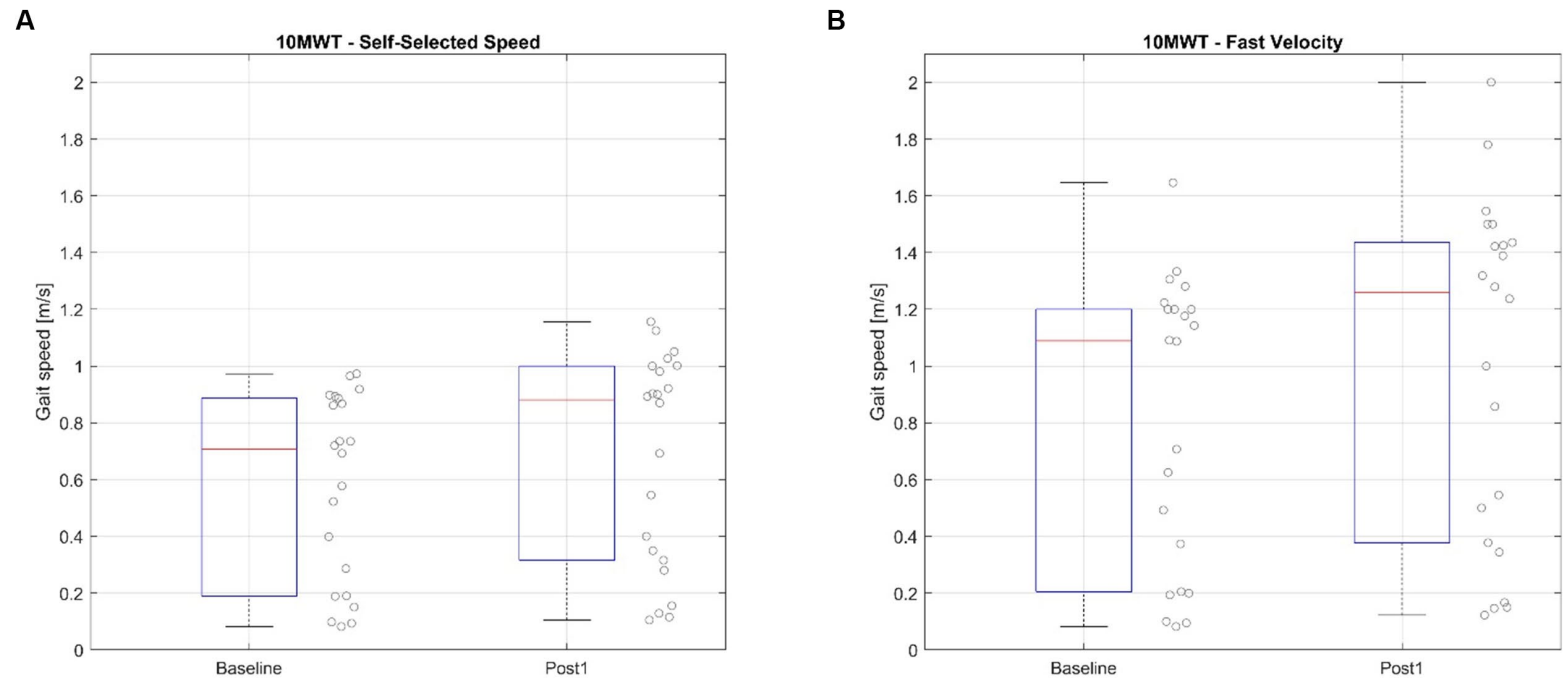
Ten Meter Walking Test before and after the BCI treatment. **(A)** Shows the values of the Self-selected speed mode, **(B)** shows the values of the Fast Velocity mode.

The results at the baseline (x̄ = 8.5 s IQR = [6.8 to 28.91]) and Post1 assessment (x̄ = 6.81 s IQR = [6.03 to 18.54]) show a significant reduction in the test time, Δ10MWT-SS [t] = −1.58 s IQR = [−2.23 to −0.49], Z = 4.901, *p* < 0.001. The results also show an increase of the test speed from the baseline (x̄ = 0.71 m/s IQR = [0.21 to 0.88]) and Post1 assessment (x̄ = 0.88 m/s IQR = [0.32 to 1]), Δ10MWT-SS [v] = 0.08 m/s IQR = [0.02 to 0.16], *Z* = 4.718, *p* < 0.001. See Figure 3A.

A non-parametric Friedman test of differences among repeated measures was conducted and rendered a Chi-squared value of 33.379 with *p* < 0.001 Therefore, a post-hoc test was conducted using the Nemenyi multiple comparison test. Table 2 presents the results.

**Table 2:** Repeated measures analysis for the 10MWT.

**10MWT**						
	**Baseline**	**S6**	**S11**	**S16**	**S21**	**Post1**
S6	0.8564	–	–	–	–	–
S11	0.3994	0.9906	–	–	–	–
S16	0.0580	0.6791	0.9767	–	–	–
S21	0.0027	0.1547	0.5620	0.9716	–	–
Post1	0.0001	0.0112	0.1015	0.5382	0.9716	–
Post2	0.0024	0.1427	0.5382	0.9657	1.000	0.9767

*Post hoc* Nemenyi test.

#### 3.2.2. Fast velocity (10MWT-FV)

The results of the 10MWT-FV in the baseline (x̄ = 5.78 s IQR = [5.15 to 26.08]) vs. Post1 assessment (x̄ = 5.14 s IQR = [4.56 to 15.09]) show a significant reduction in the test time, Δ10MWT-FV [t] = −0.99 s IQR = [−3.9 to −0.46], Z = 3.442, *p* = 0.012. The results also show an increase of the test speed from the baseline (x̄ = 1.09 m/s IQR = [0.25 to 1.2]) and Post1 assessment (x̄ = 1.26 m/s IQR = [0.41 to 1.43]), Δ10MWT-FV [v] = 0.16 m/s IQR = [0.08 to 0.3], Z = 4.649, *p* < 0.001. The mean improvement with 95% CI: Δ10MWT-FV [v] = 0.19, 95% CI [0.13, 0.25]. See Figure 3B.

A non-parametric Friedman test of differences among repeated measures was conducted and rendered a Chi-squared value of 43.12 with *p* < 0.001 Therefore, a *post hoc* test was conducted using the Nemenyi multiple comparison test.

### 3.3. Balance and gait quality

#### 3.3.1. Timed up and go

Timed Up and Go test (TUG) was also evaluated for this study. This scale asks people to stand up, walk 3 m, turn around, walk back 3 m and sit down. Patient #9 was not able to perform the TUG test before the therapy, but he could do it in 92.0 s during the Post1 assessment. This patient has been excluded from the time analysis (ΔTUG [t]). However, this patient has been included in the TUG-speed based analysis (ΔTUG [v]). The Shapiro–Wilk test was significant, so a non-parametric test was used for this comparison.

**Figure 4:**
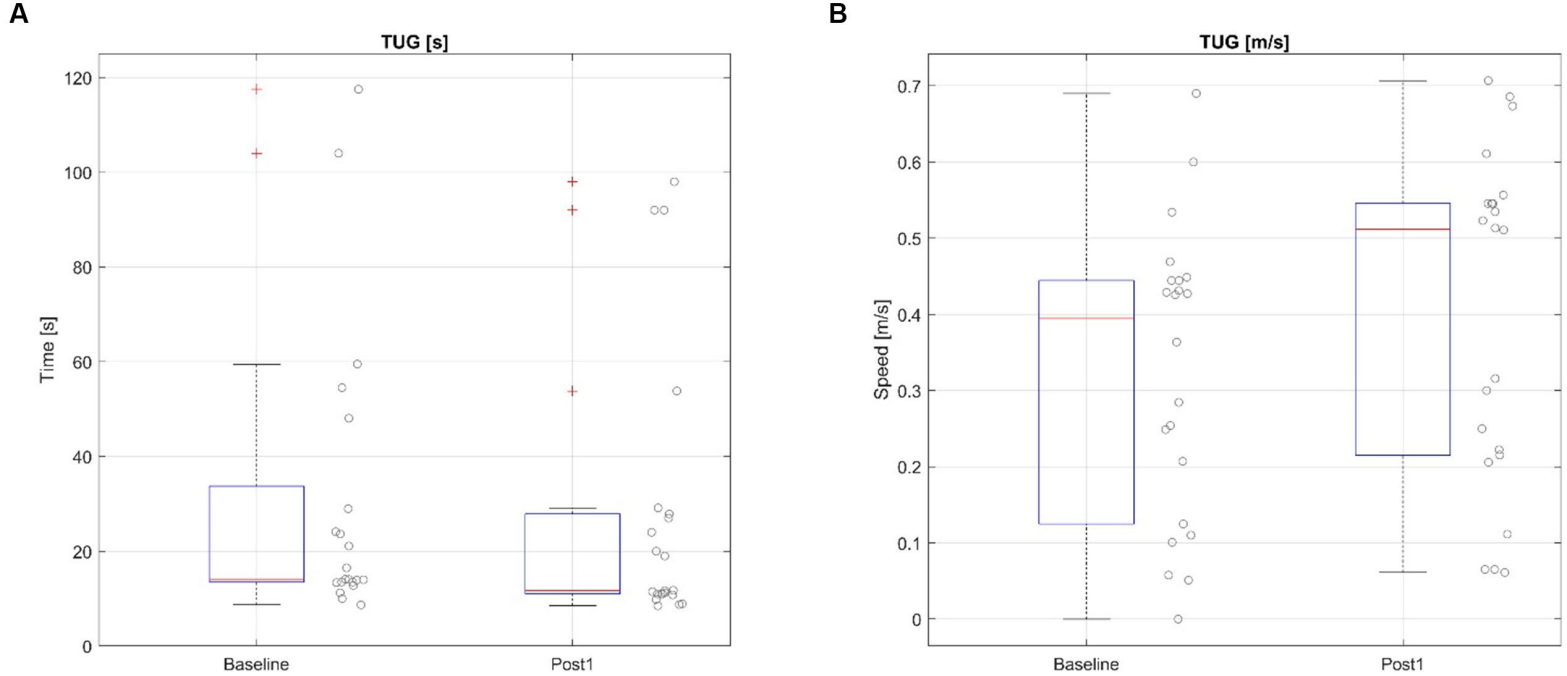
Timed Up and Go test before and after the BCI treatment based on time **(A)** and speed **(B)**.

The results in the TUG before the therapy (x̄ = 14.09 s IQR = [13.5 to 28.97]) and Post1 assessment (x̄ = 11.69 s IQR = [11–27]) show a significant reduction in the test time, ΔTUG [t] = −2.59 s IQR = [−3.62 to −1.97], Z = 4.681, *p* < 0.001. The results also show an increase of the test speed from the baseline (x̄ = 0.39 m/s IQR = [0.15 to 0.44]) to Post1 assessment (x̄ = 0.51 m/s IQR = [0.22 to 0.55]), ΔTUG [v] = 0.08 m/s IQR = [0.02 to 0.1], Z = 4.718, *p* < 0.001. See Figure 4.

A non-parametric Friedman test of differences among repeated measures was conducted and rendered a Chi-squared value of 44.415 with *p* < 0.001. Therefore, a post-hoc test was conducted using the Nemenyi multiple comparison test shown in Table 3.

**Table 3:** Repeated measures analysis for the TUG.

	**Baseline**	**S6**	**S11**	**S16**	**S21**	**Post1**
S6	0.88616	-	-	-	-	-
S11	0.02809	0.46745	-	-	-	-
S16	0.16744	0.87176	0.99452	-	-	-
S21	0.00016	0.02012	0.83997	0.42167	-	-
Post1	< 0.00001	0.00238	0.46745	0.13137	0.99703	-
Post2	0.00007	0.01120	0.74477	0.31565	1.00000	0.99959

*Post hoc* Nemenyi test.

#### 3.3.2. Functional ambulation classification

The Functional Ambulation Classification (FAC) categorizes the ambulation on different degrees of dependency. This scale ranges from 0 to 6, where 6 reflects totally independent ambulation and 0 represents the inability to walk. Scores from 0–3 indicate dependence, while 4–5 show reflect independent walking on level ground (4) or uneven surfaces (5). Only two patients’ scores on this scale changed. Subject #3 increased one point (FAC_Baseline = 5, FAC_Post1 = 6), and subject #10 increased one point (FAC_Baseline = 5, FAC_Post1 = 6). The median improvement on this scale was ΔFAC = 0 points, and IQR = [0–0.88], Z = 2.439, *p* = 0.053.

#### 3.3.3. Berg balance test

The Berg Balance Test (BBS) assess the balance on different conditions. This scale ranges from 0 to 56, where higher scores reflect better balance. The median score on this scale at the baseline was x̄ = 51.25 points, IQR = [28.25 to 54.75], where 2 patients achieve the maximum score on this scale before the therapy. These high scores during the pre-assessment suggest that this scale has an important ceiling effect limitation and that the room for improvement is relatively low. The median score on the Post1 assessment was x̄ = 52.5 points, IQR = [31.75 to 55]. The median improvement after the treatment is ΔBBS = 0 points, and IQR = [0–1.75], Z = 1.341, *p* = 0.294.

### 3.4. Range of motion and muscular balance

#### 3.4.1. Range of motion

##### 3.4.1.1. Ankle

###### 3.4.1.1.1. Flexion

The active ROM of the ankle flexion (ROMa_A_FL) at the baseline was x̄ = 5.55°, IQR = [3 to 9.18], and the median ROMa_A_FL after the therapy was x̄ = 10°, IQR = [3.7 to 15.6]. This improvement was significant, ΔROMa_A_FL = 2.13°, and IQR = [0–5.82], Z = 2.746, *p* = 0.031.

However, the passive ROM of the ankle flexion (ROMp_A_FL) at the baseline was x̄ = 15.05°, IQR = [8.59 to 21.1], and the median ROMp_A_FL after the therapy was x̄ = 19.15°, IQR = [12.92 to 22.73]. This improvement was not significant, ΔROMp_A_FL = 11.3°, and IQR = [−0.94 to 5.88], Z = −1.442, *p* = 0.281.

###### 3.4.1.1.2. Dorsiflexion

The active ROM of the ankle dorsiflexion (ROMa_A_DF) at the baseline was x̄ = 7.7°, IQR = [1.63 to 18.41], while the median ROMa_A_DF after the therapy was x̄ = 17.7°, IQR = [2.08 to 30.6]. This improvement was significant, ΔROMa_A_DF = 5.95°, and IQR = [0.45–8.64], Z = 3.598, *p* = 0.008.

The passive ROM of the ankle dorsiflexion (ROMp_A_DF) at the baseline was x̄ = 24.75°, IQR = [19.35 to 31.26], and the median ROMp_A_DF after the therapy was x̄ = 30.05°, IQR = [21.33 to 37.25]. This improvement was significant, ΔROMp_A_DF = 4.32°, and IQR = [1.16–10.09], Z = −3.368, *p* = 0.023.

##### 3.4.1.2. Knee

###### 3.4.1.2.1. Flexion

The active ROM of the knee flexion (ROMa_K_FL) at the baseline was x̄ = 114.6°, IQR = [109.2 to 118.1], and the median ROMa_K_FL after the therapy was x̄ = 117.1°, IQR = [112 to 124.6]. This improvement was not significant, ΔROMa_K_FL = 2.5°, and IQR = [−1.1 to 9.5], Z = 1.929, *p* = 0.126.

The passive ROM of the knee flexion (ROMp_K_FL) at the baseline was x̄ = 128.9°, IQR = [124.1 to 133.95]. The median ROMp_K_FL after the therapy was x̄ = 135.3°, IQR = [126.6 to 139.7]. This improvement was not significant, ΔROMp_K_FL = 2.95°, and IQR = [−1 to 9.25], Z = 2.572, *p* = 0.043.

#### 3.4.2. Modified Ashworth scale

The spasticity in the ankle at the baseline was x̄ = 3 points, IQR = [1 to 3], which changed to x̄ = 2.5 points, IQR = [1–3] in the Post1. This improvement was significant, ΔMAS_ankle = 0 points, and IQR = [−1 to 0], Z = 2.656, *p* = 0.038.

#### 3.4.3. Manual muscle test

##### 3.4.3.1. Ankle

The Manual Muscle Test (MMT) of the ankle flexion (MMT_A_FL) at the baseline was x̄ = 6 points, IQR = [4.12 to 8.38]. In the Post1, it was x̄ = 8.5 points, IQR = [5–9]. This improvement was significant, ΔMMT_A_FL = 0.25 points and IQR = [0–1], Z = 2.907, *p* = 0.024.

The MMT of the ankle dorsiflexion (MMT_A_DF) at the baseline was x̄ = 6 points, IQR = [4–7], and in the Post1 was x̄ = 7 points, IQR = [4.5 to 9]. This improvement was significant, ΔMMT_A_DF = 0.73 points and IQR = [0–1], Z = 2.763, *p* = 0.031.

##### 3.4.3.2. Knee

The Manual Muscle Test (MMT) of the knee flexion (MMT_K_FL) at the baseline was x̄ = 9 points, IQR = [8–10], and was x̄ = 10 points, IQR = [9–10] in the Post1. This improvement was not significant, ΔMMT_K_FL = 0.34 points and IQR = [0–1], Z = 2.33, *p* = 0.068.

The MMT of the knee extension (MMT_K_EX) at the baseline was x̄ = 8.25 points, IQR = [6.25 to 9]. In the Post1, it was x̄ = 9 points, IQR = [8–10]. This improvement was significant, ΔMMT_K_EX = 0.68 points and IQR = [0–1], *Z* = 2.777, *p* = 0.031.

### 3.5. Motor function of upper and lower limbs

The motor function of the upper and lower extremities was assessed using the motor section of the Fugl-Meyer Assessment (FMA). The FMA for the upper extremities (FMA-UE) ranges from 0 to 66 points, while the FMA for the lower extremities (FMA-LE) ranges from 0 to 36 points.

The FMA-UE of the upper extremity at the baseline was x̄ = 22.75 points, IQR = [16.25 to 32.75]. It was x̄ = 24 points, IQR = [18–37] in the Post1. This improvement was not significant, ΔFMAue_m = 0.75 points and IQR = [−0.38 to 5.62], Z = 2.115, *p* = 0.103.

The FMA-LE of the upper extremity at the baseline was x̄ = 24 points, IQR = [20.5 to 25.5]. In the Post1, it was x̄ = 25 points, IQR = [22–27]. This improvement was not significant, ΔFMAle_m = 1 points and IQR = [0–2.5], Z = −1.872, *p* = 0.166.

### 3.6. Cognition and daily living activities

#### 3.6.1. Barthel index

The score for daily living activities assessed by the Barthel Index (BI) at the baseline was x̄ = 90 points, IQR = [80–90]. In the Post1, the BI was x̄ = 90 points, IQR = [81.25 to 95]. This improvement was significant, ΔBI = 1.25 points and IQR = [0–5], Z = 3.078, *p* = 0.021.

#### 3.6.2. Montreal cognitive assessment

Cognitive abilities were assessed by the Montreal Cognitive Assessment. This test contains 7 sections: Visual-execution (Vis-Exe), Abstraction (Abst), Attention (Att), Delayed recall (DRec), Language (Lang), Naming (Nam) and Orientation (Ori). The total score of the MOCA scale (MOCA_total) at the baseline was x̄ = 26 points, IQR = [22.88 to 28.25], and changed to x̄ = 27 points, IQR = [24–30] in the Post1. This improvement was not significant, ΔMOCA = 0.5 points and IQR = [−0.5 to 1.62], Z = 1.959, *p* = 0.126.

#### 3.6.3. Stroop color word test

Cognitive state was also assessed by the Stroop Color Word test (SCWT). This test contains three different sub-test or cards: the Word card, the Color card and the Color-Word card.

The Word card score at the baseline was x̄ = 73 points, IQR = [46–96], and in the Post1 was x̄ = 80 points, IQR = [47–98]. This improvement was not significant, ΔSCWT_Word = 3 words and IQR = [−1 to 6], *Z* = −2.798, *p* = 0.044.

The Color card score at the baseline was x̄ = 68 points, IQR = [48–93]. In the Post1, it was x̄ = 65 points, IQR = [50–92]. This improvement was not significant, ΔSCWT_Color = 1 word and IQR = [−2 to 6], Z = −1.171, *p* = 0.383.

The Color-Word card score at the baseline was x̄ = 24 points, IQR = [15.5 to 30], and was x̄ = 26 points, IQR = [18–34] in the Post1. This improvement was significant, ΔSCWT_ColorWord = 1.5 words and IQR = [0–6], *Z* = −3.28, *p* = 0.024.

### 3.7. Functional outcomes in the long term

The analysis of the long-term effects is based on comparisons between the Post1 vs. Post2 and Post1 vs. Post3 assessments. Table 4 shows the scales that shown a significant results in the statistical analysis.

**Table 4:** Summary of the changes in tests of long-term functional outcomes that showed statistical significance.

**Scale**	***N***	**Post1 vs. Post2** **Median [IQR]**	***p***	***n***	**Post1 vs. Post3** **Median [IQR]**	***p***
FMA-UEm	22	1.5 [0 to 3]	0.021	15	4 [3 to 7]	0.021
MMTAnkle FL	22	0 [0 to 0]	0.245	15	1 [0 to 1]	0.046
TUG	22	0.03 [−0.61 to 0.97]	0.704	14	1.61 [0.68 to 3.34]	0.021
10MWTSS	22	0.06 [−0.29 to 0.91]	0.5	15	0.64 [0.05 to 1.19]	0.038

#### 3.7.1. Middle-term effects

The only significant change seen in the middle-term was in the functionality of the upper limb assessed by the Fugl Meyer Assessment, ΔFMAue_m = 1.5 points and IQR = [0–3], Z = 2.115, *p* = 0.021. This change shows that the therapy can help the patients improve the motor ability of the hand during the first month after the therapy.

#### 3.7.2. Long-term effects

Seven patients did not complete the Post3 assessment. Five of them did not come to the Post3 assessment due to lack of motivation, and two of them had personal and logistical issues that prevented them from coming to the therapy center. Therefore, the analysis of the long-term effects (Post3) is presented using the data from 15 patients.

The functional evaluation 6-months after the therapy shows an increase of the FMAue scale, ΔFMAue_m = 4 points and IQR = [3–7], *Z* = 2.115, *p* = 0.021.

The comfortable gait speed also increased significantly 6 months after the therapy, Δ10MWT-SS = 0.64 m/s and IQR = [0.05–1.19], Z = 4.901, *p* = 0.038.

### 3.8. BCI performance

**Figure 5:**
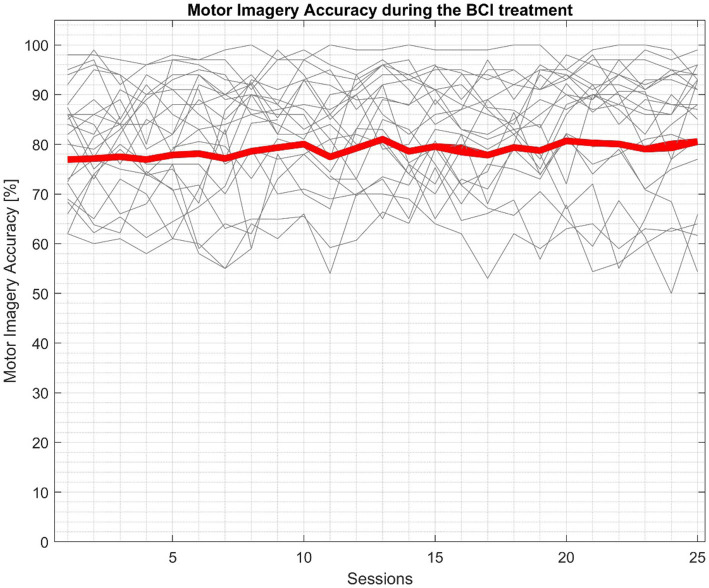
BCI performance during the BCI therapy. Gray lines represent the participants accuracy during the BCI treatment of each session, while the red line shows the mean accuracy of all participants.

The BCI performance was evaluated using the motor imagery accuracy provided by the system after each session. Figure 5 shows the evolution of the motor imagery accuracy during the treatment for each patient (gray lines). The mean MI accuracy for all participants was 82.68% (SD = 10.05).

### 3.9. Adverse events

No adverse events have been reported during the study.

## 4. Discussion

This study shows that 22 chronic stroke patients with a median time since stroke onset of 4 years improved their walking speed by 0.19 m/s 95% CI [0.13, 0.25] on average. All of these patients performed 18.75 h of BCI based treatment for the lower limb in a sitting down position.

The results show that patients significantly improved their gait speed assessed by the 10MWT. This improvement is above the substantial meaningful change, 0.14 m/s (Perera et al., 2006). Results also showed that treatment increase patients’ performance of daily living activities, improved their cognitive skills, reduced spasticity in the ankle and increased the ankle range of motion and muscular strength of the main joints involved into the gait patterns.

The functional improvement achieved after the BCI training was maintained 1 month after the end of the therapy. Middle-term effects were assessed in the Post2 assessment, 1 month after the last BCI session. Patients reported an increase of the upper extremity function assessed by the FMA-UE scale. This improvement contributed to the increased participation of the subjects in the daily living activities. The upper extremity functionality increases continued at least until the long-term assessment, 6 months after the end of the therapy (Post3). Patients also reported an increase of the comfortable gait speed assessed by the 10MWT-SS.

Based on the repeated measures analysis of the 10MWT, patients started showing a significant improvement of gait speed after session 21. Therefore, the protocol based on 25 sessions distributed in 3 times per week seems to be a viable treatment schedule, although further exploration of different treatment schedules could identify more effective approaches.

On one hand, a large meta-analysis done by Mehrholz et al. (2018) shows that treatment based on electromechanical gait devices with end effector (EGAIT_EE) seems to be one of the most powerful approaches for the gait rehabilitation after stroke. Those devices are able to reproduce walking patterns with high accuracy, and patients can train their gait with different levels of body weight support. Pohl et al. (2007) conducted a randomized clinical trial including 170 patients in the acute state (less than 60 days since the stroke onset). This study reported that patients in the EGAIT_EE group (*n* = 77) increased gait speed 0.31 m/s (SD = 0.40), while patients in the control group improved only 0.18 m/s (SD = 0.28). This big improvement occurred because the treatment was given in a very early stroke stage and the baseline gait velocity was substantially lower than the mean gait speed of this study. The same reasoning can explain the results from other clinical trials like Werner et al. (2002), Tong et al. (2006), Chua et al. (2016), Aprile et al. (2019), and Kim et al. (2019). Nevertheless, other studies such as Peurala et al. (2005) included patients in the chronic stage and obtained results similar to our study; the change in the gait speed was 0.11 m/s (SD = 0.05) in the EGAIT_EE group.

On the other hand, other BCI devices reported similar improvements in gait speed with the same target population. Chung et al. (2020) carried out a clinical trial with both arms to compare the BCI-based treatment to the functional electrical stimulation (FES) treatment alone. Results show that patients in the BCI group improved by 0.13 m/s (SD = 0.03) while the improvement in the control group was about 0.05 m/s (SD = 0.04). Mihara et al. (2021) had a similar approach with a two-arm clinical randomized trial. Fifty-four patients were recruited for this study, with 28 in a real feedback group and 26 in the sham feedback group. The results demonstrated that patients in the experimental group significantly increased their gait speed by 0.10 m/s (SD = 0.08), and patients in the sham group 0.07 m/s (SD = 0.06). Finally, Mrachacz-Kersting et al. (2016) reported the highest improvement in gait speed in the control 0.32 m/s (SD = 0.33) and experimental groups 0.49 m/s (SD = 0.55). This big improvement could be explained because 12 out of 24 participants were not able to perform the gait test before the treatment, but 8 of them could perform it after the last session. This fact also explains the high SD in both groups.

Most of the gait rehabilitation techniques assumed that patients should be standing when retraining their gait patterns. Usually, those techniques require a system for supporting the user’s weight, especially in moderate or severely impaired patients. Other systems that use functional electrical stimulation also recommend the stimulation during walk. The BCI device used in this study (recoveriX, g.tec medical engineering GmbH) does not need a body weight support system because patients are seated during the BCI training. This is a safer approach because it reduces the risk of falling, but patients can still train gait patterns and thereby increase functionality, gait speed and coordination and balance. The role of neurofeedback provided through BCI technology seems to be key in the rehabilitation process.

Another discussion point is the low sensitivity of the FMA-LE to detect functional changes in the lower extremity. The FMA-LE functional scale is oriented to evaluate specific movements of the lower extremity and not the walking ability. From the functionality point of view, the main task of the lower extremity is walking, this fact leads researchers to question the sensitivity of the FMA-LE scale for the detection of changes in gait ability in stroke patients. All the gait scales used in this protocol showed significant changes, but this contrasts with the lack of significant results obtained on the FMA-LE scale.

There were no significant changes in the MOCA scale. This result may be explained in part by the exclusion criteria. All participants had a MOCA score of 22 or higher, and they did not have major cognitive deficits.

The results involving the spasticity in the knee (MAS_Knee) and balance (BBS) were not significant. This is because most of the study participants did not show spasticity in the knee before the study or the values in the BBS at the baseline were high. Finally, the FAC assessment was not sensitive enough to detect changes in the gait for this population.

The long-term results shown in Table 4 are also clinically relevant to understand the progression of rehabilitation at a later stage. Our results show that stroke patients continued to improve their functional abilities even 6 months after therapy. Patients improved their gait, gained more independence for other complex tasks, and increased their participation in society – all of which probably contributed to this ongoing improvement. It is also important to note the positive long-term effect on the upper limb. Patients showed significant improvement in upper extremity function 1 month after therapy and continued to improve 6 months after therapy. This is a clear example of how the positive effect of gait rehabilitation has a positive impact on whole-body rehabilitation.

The BCI performance plot indicates that participants were able to control the system well, which fits well to the work of Ang et al. (2011). The MI accuracy reflects the degree of stimulation that the system delivered to the user. Therefore, given the mean MI accuracy across all participants (82.68%), the therapy was highly successful in terms of the FES and VR dosage.

This study has some limitations. First, the patients involved in this study were in the chronic stage of stroke recovery, which may limit the generalizability of the results to the subacute phase. Previous research has shown that the time since stroke onset is a crucial factor for functional rehabilitation, and that patients in the subacute phase tend to improve gait functionality more than patients in the chronic stage. Second, this study did not have a control group, which prevented a direct comparison to a sham condition using the same methods. A control group would have allowed us to rule out the effects of spontaneous recovery, motivation, attention, and placebo on the outcomes of the BCI therapy.

No adverse events were reported during the investigation. This is also consistent with the studies mentioned above that use BCI devices for stroke rehabilitation. The customization of the FES settings for each individual, the patient’s safe (seated) position during the therapy, the short session time and the easy set-up of this BCI system help explain that patients did not have side effects.

The clinical investigation has been conducted in accordance with the GCP.

## Data availability statement

The datasets presented in this article are not readily available because patients’ data need to be treated according to current data protection laws and ethical guidelines. Requests to access the datasets should be directed to CG, guger@gtec.at.

## Ethics statement

The studies involving humans were approved by Ethikkommission des Landes Oberösterreich (Nr. 1,126/2020). The studies were conducted in accordance with the local legislation and institutional requirements. The participants provided their written informed consent to participate in this study. Written informed consent was obtained from the individual(s) for the publication of any potentially identifiable images or data included in this article.

## Author contributions

MS-R: Data curation, Formal analysis, Investigation, Methodology, Writing – original draft. WC: Investigation, Writing – review & editing. RO: Supervision, Writing – review & editing. SS: Data curation, Writing – review & editing. TO: Writing – review & editing. KK: Writing – review & editing. SL: Writing – review & editing. BA: Writing – review & editing. CG: Supervision, Writing – review & editing.
